# Exposure to Atmospheric Ultrafine Particles Induces Severe Lung Inflammatory Response and Tissue Remodeling in Mice

**DOI:** 10.3390/ijerph16071210

**Published:** 2019-04-04

**Authors:** Yara Saleh, Sébastien Antherieu, Romain Dusautoir, Laurent Y. Alleman, Jules Sotty, Corentin De Sousa, Anne Platel, Esperanza Perdrix, Véronique Riffault, Isabelle Fronval, Fabrice Nesslany, Ludivine Canivet, Guillaume Garçon, Jean-Marc Lo-Guidice

**Affiliations:** 1 EA4483-IMPECS, Institut Pasteur de Lille, Université de Lille, CHU Lille, 59045 Lille CEDEX, France; yara.saleh@univ-lille.fr (Y.S.); sebastien.antherieu@univ-lille.fr (S.A.); romain.dusautoir@univ-lille.fr (R.D.); jules.sotty@univ-lille.fr (J.S.); corentin.de-sousa@univ-lille.fr (C.D.S.); anne.platel@pasteur-lille.fr (A.P.); fabrice.nesslany@univ-lille.fr (F.N.); ludivine.canivet@univ-lille.fr (L.C.); guillaume.garcon@univ-lille.fr (G.G.); 2 Département Sciences de l’Atmosphère et Génie de l’Environnement (SAGE), IMT Lille Douai, Université de Lille, 59000 Lille, France; laurent.alleman@imt-lille-douai.fr (L.Y.A.); esperanza.perdrix@imt-lille-douai.fr (E.P.); veronique.riffault@imt-lille-douai.fr (V.R.); isabelle.fronval@imt-lille-douai.fr (I.F.)

**Keywords:** Ultrafine particles, mice, (sub)chronic exposure, inflammation, lung tissue remodeling

## Abstract

Exposure to particulate matter (PM) is leading to various respiratory health outcomes. Compared to coarse and fine particles, less is known about the effects of chronic exposure to ultrafine particles, despite their higher number and reactivity. In the present study, we performed a time-course experiment in mice to better analyze the lung impact of atmospheric ultrafine particles, with regard to the effects induced by fine particles collected on the same site. Trace element and PAH analysis demonstrated the almost similar chemical composition of both particle fractions. Mice were exposed intranasally to FF or UFP according to acute (10, 50 or 100 µg of PM) and repeated (10 µg of PM 3 times a week during 1 or 3 months) exposure protocols. More particle-laden macrophages and even greater chronic inflammation were observed in the UFP-exposed mice lungs. Histological analyses revealed that about 50% of lung tissues were damaged in mice exposed to UFP for three months versus only 35% in FF-exposed mice. These injuries were characterized by alveolar wall thickening, macrophage infiltrations, and cystic lesions. Taken together, these results strongly motivate the update of current regulations regarding ambient PM concentrations to include UFP and limit their emission.

## 1. Introduction

According to a new study published by Lelieveld et al. (2019), air pollution leads to 8.8 million extra early deaths a year [1]. These data exceed the last estimations of the World Health Organization [2]. Particulate matter (PM) is a main component of the atmospheric pollution. In October 2013, outdoor air PM was classified by the International Agency of Research on Cancer (IARC) as carcinogenic to humans (Group I) [3]. Numerous epidemiological and experimental studies show that PM has adverse health effects, such as pulmonary and cardiovascular diseases [4]. In particular, it has been shown that ambient air pollution is associated with an increased risk of chronic obstructive pulmonary disease (COPD), asthma, and lung cancer mortality [5,6,7]. 

PM may be characterized by its chemical composition, which plays an important role in its toxicity. The chemical characteristics of PM depend on emission sources, atmospheric conditions, location as well as on the interactions with other pollutants such as gases, polycyclic aromatic hydrocarbons (PAH) or metals, leading to the formation of new toxic compounds [8]. Additionally, physical factors like size and morphology play a key role in the toxicity of particles by enabling them to penetrate more or less deeply into the respiratory tract [8,9,10,11,12,13]. The size of particles is defined by their aerodynamic equivalent diameter (AED) and three particulate fractions are usually distinguished: coarse (PM_10_, AED ≤ 10 µm), fine (PM_2.5_, AED ≤ 2.5 µm), and ultrafine particles (PM_0.1_ or UFP, AED ≤ 0.1 µm). This latter fraction is the most abundant in urban and industrial environments in terms of number concentrations with contributions that can reach more than 80% of the total airborne particle number [14,15]. The largest contributors to the emission of UFP are road traffic, combustion of biomass and fossil fuels, industrial emissions and more recently the use of nanotechnology [16,17]. In addition, UFP can be either emitted directly to the atmosphere (primary) or formed secondarily in the atmosphere from precursor gaseous pollutants (secondary) [18].

UFP are described as potentially more harmful than larger particles due to (i) their higher surface area per unit of mass, increasing the amount of adsorbed toxic materials potentially available, and the exchange surface with the physiological medium, and (ii) their lower aerodynamic diameter allowing them to penetrate into the deepest areas of the respiratory system, cross the alveolar barrier and translocate to other organs through the systemic circulation [19,20,21]. Moreover, UFP are thought to be eliminated less efficiently from lung, which increases their residence time and facilitates their accumulation in the respiratory tract [22]. 

Several epidemiological studies have suggested the link between exposure to UFP and increase in hospital admissions [23,24]. These studies have also shown that exposure to these particles exacerbates different diseases such as asthma, and COPD [25,26,27]. The lungs are in direct contact with UFP; however, the small size of these particles allows them to reach the blood circulation and spread to other organs; therefore, the effects of UFP are not limited to the lungs but can be observed elsewhere such as in cardiovascular and metabolic diseases [28,29].

As UFP are not as well studied and characterized as PM_10_ and PM_2.5_, their toxic effect is potentially underestimated and has not yet been taken into consideration by the air quality regulations aiming to reduce the emissions of PM and lower their impact on health. Most of the current studies concerning nanomaterials focus on the toxicity of manufactured nanoparticles (MNP), but not on environmental UFP. The results obtained in these studies, highlighting the significant health effects of MNP, raises interest in studying the toxicity of UFP to which everyone is exposed on a daily basis. Consequently, more experimental studies are needed in order to better understand the cellular and molecular mechanisms involved in the toxicity of chronic exposure to UFP. In this study, we performed a time-course experiment on a mouse model (BALB/c) to analyze the effect of short and long-term exposures to atmospheric ultrafine (with an AED < 0.18 µm) and fine (with an AED between 0.18 and 2.5 µm) particles collected from the same urban-industrial site. The main objective was to assess the pulmonary toxicity of ultrafine atmospheric particles, and, secondarily to analyze it with regard to that induced by fine particles.

## 2. Materials and Methods 

### 2.1. PM Sampling

Fine and ultrafine particles (respectively defined in the present study as PM_0.18-2.5_ or fine fraction – FF, and PM_0.18_ – UFP) were collected at an urban background site in Grande-Synthe, approximately 2 km south of the Dunkirk industrial harbor area (North of France). Sampling was conducted on a weekly basis from 9 September 2013 to 14 April 2014 (26 weeks) using a High-Volume Impactor Sampler (HVIS, flow rate of 400 L/min) to collect FF by impaction on Teflon stripes and UFP by filtration on 8” × 10” polycarbonate (PC) membranes [30]. The cumulated FF corresponding to 26 weeks was transferred with a Teflon tip into an acid-cleaned dry vial, weighed and stored along with all the UFP PC membranes at −20 °C until chemical analyses, handling for extractions and toxicological assays. The UFP of Dunkirk represent 10.5% of the PM_2.5_ fraction mass estimated over the 26 weeks sampling period. This is higher than the usual values (< 5%) found in ambient air probably because it includes particles slightly larger than the typical ultrafine faction (below 100 nm) and because the impactors were located near both large industries and traffic roads [31]. 

Simultaneous to the FF and UFP collection by HVIS, a 13-stage cascade impactor (Dekati; flow rate of 30 L/min) collecting different size fractions (10.5; 6.7; 4; 2.5; 1.65; 1; 0.65; 0.4; 0.26; 0.17; 0.1; 0.06 and 0.03 μm) on pre-weighted 25 mm-diameter Teflon filters was deployed at the same site. This additional sampling allowed to better characterize the chemical composition temporal variability and helped to identify the main sources of particles impacting the sampling site. A hierarchical clustering analysis (HCA) carried out on the UFP samples resulted in five sources originating from industrial activities (steel industry, metal processing), road and maritime traffic and road soil resuspension. This also enabled to validate and determine precisely the mass of UFP collected with the HVIS, as large PC membranes are not suitable for gravimetric analysis. UFP were transferred in a Hanks’ Balanced Salt Solution (HBSS; ThermoFisher Scientific, Illkirch, France). Filters were handled in an ultra-clean laboratory using acid-washed plastic and glassware to prevent both biological and trace element contaminations. The 26 large PC membranes used to collect the UFP were cut in nine smaller parts each, and each part was weighted. For each large membrane, one part was kept for chemical analysis, while the remaining eight parts were quantitatively transferred into an HBSS solution by three successive water-cooled ultrasonic extractions (Vibracell 75455, 500 W, 20 KHz). All the 26 membranes were combined in one bulk sample of suspended UFP in HBSS. For the precise determination of the UFP mass in the bulk sample, we relied on both (i) the estimation of the mass of UFP on each membrane (assuming the same constant weight for clean non-exposed membranes), and (ii) the chemical composition of the UFP (PM_0.18_) collected with the HVIS compared to the one collected with the Dekati low-volume sampler (PM_0.03–0.26_). Plotting the 26 weekly data points from both high and low volume samplers corresponding to the mass concentrations of chemical elements, we obtained a linear regression allowing to calculate the ratio between the mass of UFP (PM_0.18_) to be determined with the mass of UFP (PM_0.03–0.26_) weighted on 25 mm Teflon filters. The obtained suspension of UFP in HBSS (2.7 µg/µL) was then kept at −20 °C until further use.

### 2.2. Particle Size Distribution

The hydrodynamic diameter of the FF and UFP was measured after suspending particles in HBSS (1 mg/mL) using a Dynamic Light Scattering (DLS) Zetasizer nano ZS (Malvern Instruments, Orsay, France). Measurements of the zeta potential carried out at 25 °C showed negative values for both fractions, of −17.1 ± 0.3 mV for UFP and −12.7 ± 0.3 mV for FF. According to Canepari et al. (2013), these values correspond to a moderate degree of repulsion among particles (i.e. when the absolute value of the zeta potential is > 30 mV the dispersion resist aggregation, whereas when it is null the particles flocculate) [32]. Over the five-min long repeated measurements, there was no evidence of an unstable state of the suspensions, suggesting a good stability. In addition, the particle suspensions were mechanically shaken and sonicated just before exposing the mice in order to ensure a good homogenization.

### 2.3. Trace Elements and PAH Analysis in FF and UFP

Trace elements concentrations were measured in three aliquots of FF (1.2 ± 0.4 mg) after acid digestion in Teflon reactors with ultrapure reagents (HNO_3_; HF; H_2_O_2_) in a microwave oven (Milestone ETHOS, ThermoFisher Scientific, Courtaboeuf, France) for 20 min at 200 °C [33]. The digests were transferred to clean polypropylene tubes and diluted to 50 mL with ultrapure water. The detailed methodology and quality control measurements for total metal concentration analysis (blanks, detection limits, uncertainties) were reported previously [34]. Trace elements concentrations (Al, As, Ba, Be, Ca, Cd, Ce, Co, Cr, Cs, Cu, Fe, K, La, Mg, Mn, Mo, Na, Ni, Pb, Rb, Sb, Sn, Sr, Tl, V, Zn) were determined in triplicates by ICP-MS (NeXion 300x, Perkin Elmer, Waltham, MA, USA). Measurements of blanks and quality control (QC at 0.4 µg/L) of multielement standard solutions were performed repetitively during analytical runs. An internal standard (^69^Ga, ^103^Rh) was added (1 µg/L) to all the analyzed solutions to correct for the drift of the ICP-MS signal. In addition, several samples of a standard reference material (1.1 ± 0.5 mg; NIST SRM 2584 particles, Indoor Dust) were used to validate the extraction procedure.

Pressurized liquid extraction of PAHs in the FF samples was realized in acetonitrile with a Dionex ASE 200 instrument (ThermoFisher Scientific) in a stainless-steel 11-mL cell containing the particles to be extracted (i.e., 5.13 mg). The extract was gently evaporated for enrichment under a stream of nitrogen in a TurboVap II water bath kept at 60 °C (Zymark, Roissy, France) to a final volume of 500–1000 µL. The analytical methodology, detailed elsewhere [35], use a high-pressure liquid chromatography (HPLC) consisting of a Waters 2695 Alliance system (Waters SA, Saint-Quentin-en-Yvelines, France) coupled to an on-line 996-photodiode array and a 2475-fluorimetric detector. Recovery efficiencies and analytical detection limits were estimated for each PAH using a certified material (i.e., NIST SRM-1649a, Urban Dust). The method allowed for the determination of the concentrations of 18 PAHs: Fluoranthene, Pyrene, Benzo(c)phenanthrene, Benzo(a)anthracene, Chrysene, 5-Methylchrysene, Benzo(e)pyrene, Benzo(b)fluoranthene, Benzo(j)fluoranthene, Benzo(k)fluoranthene, Benzo(a)pyrene, Dibenzo(a,l)pyrene, Dibenzo(a,h)anthracene, Benzo(g,h,i)perylene, Indeno(1,2,3-c,d)pyrene, Dibenzo(a,e)pyrene, Anthanthrene and Coronene. 

Regarding the UFPs, due to their smaller quantity, chemical analyses were performed directly on the bulk suspension in HBSS. The analytical procedure for trace element analysis by ICP-MS was performed on four aliquots of the bulk suspension in HBSS equivalent to 600 µg of UFPs after acid digestion following the same procedure as described for the FF. For PAH analysis, 5.37 mg of UFPs from the HBSS suspension were deposited onto a pre-baked quartz fiber filter (∅ = 47 mm, Tissuquartz 2500QAT-UP Pallflex, VWR, Fontenay-sous-Bois, France) then extracted twice and analyzed in triplicates using the same procedure as previously described.

### 2.4. Animal Housing 

SOPF (specific and opportunistic pathogen free) male BALB/c mice nine weeks of age were obtained from Janvier Labs (Le Genest-Saint-Isle, France). This strain is known to be relatively sensitive to chemical induction of lung tumors [36]. Mice were group-housed in ventilated and isolated cages under specific pathogen-free conditions and fed with autoclaved food. All animal procedures were approved by the local Animal Ethics Committee (approval number: 2015120411561967). Mice were monitored every day to assess their health, well-being, body weight, external physical appearance, clinical signs, and changes in behavior.

### 2.5. Animal Exposure to Particles

**Figure 1:**
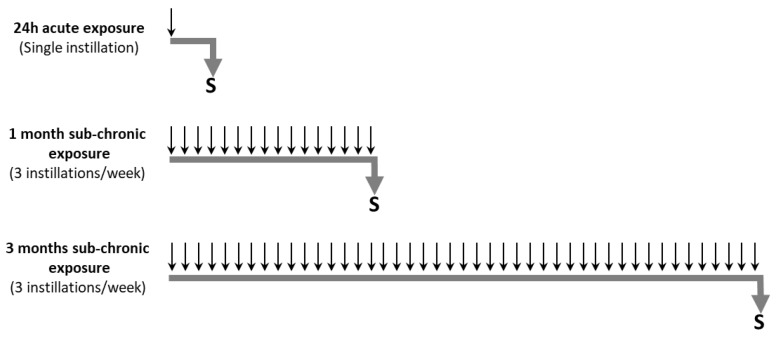
Experimental protocol of acute and subchronic exposures of mice to FF and UFP. Particles were suspended in 40 µL of HBSS. In the acute exposure, mice were sacrificed (S) 24 h after receiving a single dose of particles (10, 50 or 100 µg). For the subchronic exposure, the mice were exposed 3 times a week for 1 or 3 months to 10 µg of particles. Control mice received HBSS without particles. Arrows pointing down mean intranasal instillations of a dose of HBSS, FF or UFP.

Particle suspensions were sonicated prior to use. After anesthesia with 2% isoflurane, the mice received intranasally 40 µL of HBSS (Control group), or different doses of FF or UFP suspended in 40 µL HBSS according to three exposure protocols: acute (24 h), or subchronic exposures for 1 month or 3 months. During the acute exposure, the mice (5 mice/group) received a single dose of 10 µg, 50 µg or 100 µg of FF or UFP; during the subchronic exposures, mice (8 mice/group: 5 mice used to prepare lung homogenates and 3 mice destined to histological analyses) received 10 µg of FF or UFP for 1 or 3 months, at 3 instillations per week (Figure 1). 

### 2.6. Biological Sample Collection

The mice were sacrificed 2 hours after receiving the last instillation. Bronchoalveolar lavage (BAL) (n = 5) was collected under anesthesia with 10 µL/g of Ketamine/Xylazine mix (100 mg/kg ketamine and 20 mg/kg Xylazine) administered intraperitoneally. An endotracheal cannula was installed surgically and two lavages were carried out with 2 × 1 mL of sterile Phosphate-buffered saline (PBS; ThermoFisher Scientific, Illkirch, France). Afterwards, the lungs were harvested. One section was stored in RNA Later (Ambion,ThermoFisher Scientific, Illkirch, France) for qPCR analysis and another part frozen in liquid nitrogen and then stored at −80 °C until further analyses.

For histological analyses, another group of mice (n = 3) was used. The lungs were fixed by intratracheal instillation of 4 % (*v*/*v*) paraformaldehyde in PBS, immersed in this fixative solution overnight, and then embedded in paraffin.

### 2.7. Numeration of BAL Cells

Once the BAL fluid was collected (2 × 1 mL), it was immediately kept on ice and then centrifuged at 3000 g for 10 min at 4 °C. The obtained cell pellets were pooled together and suspended in 500 µL of PBS. Cell viability in BAL was determined by Trypan blue exclusion test. Smears for cell differentiation were prepared by cytocentrifugation (cytospin-2, Shandon Products Ltd., ThermoScientific, Illkirch, France) on a superfrost glass slide. Triple-blind differential cell count was performed by microscopy on cytospin slides after staining with May–Grünwald–Giemsa (Sigma Aldrich, Saint-Quentin-Fallavier, France). The percentage of polynuclear neutrophils (PNN) and macrophages was calculated after counting at least 200 cells in at least four randomly selected fields on each slide. 

### 2.8. Lung Histology and Lung Injury Scoring

Paraffin-embedded lung tissue samples were used to prepare hematoxylin and eosin (HE)-stained slides in order to perform histologic analysis (scores detailed in Table 4). Lung tissue sections were also stained with Sirius red (Sigma Aldrich) in order to search for fibrotic lesions Three 5 µm serial sections separated by 100 µm were realized for HE and Sirius staining.

For immunohistochemistry, a rabbit monoclonal Anti-F4/80 antibody (SP115; Abcam, Paris, France) was used to stain macrophages on lung tissue sections. An HRP anti-rabbit (Abcam) was used as a secondary antibody. 

The slides were observed by a veterinary pathologist blinded to the study with an optical microscope Eclipse Ni with DS-Ri2 camera (Nikon, France). As the lungs were formalin fixed and paraffin embedded, whole parenchymal collapse might occur. Thus, for alveolar wall thickness evaluation, a comparison with the erythrocytes size was performed. Moreover, to assess the extent of lung tissue damage, a ratio between the lesioned part of each section and the whole section surface was calculated.

### 2.9. RNA Extraction and Real-Time PCR

Total RNAs were extracted from RNA later treated tissue samples using the miRNeasy mini kit (Qiagen, Courtaboeuf, France) according to the manufacturer’s protocol. The RNA concentration was measured with the Biospecnano spectrophotometer (Shimadzu, Marne-la-Vallée, France). RNA quality was determined with the Experion™ Automated Electrophoresis System (Biorad, Marnes la Coquette, France). The RNA samples were reverse-transcribed to complementary DNA (cDNA) using the high capacity cDNA reverse transcription kit (Applied Biosystems, Courtaboeuf, France). The expression of genes involved in PAH metabolism (Cyp1a1, Mm00487218_m1; Cyp1b1, Mm00487229_m1) and inflammation (Il-6, Mm00446190_m1; Il-1b, Mm00434228_m1 and Il-10, Mm01288386_m1) was measured in duplicate wells by comparative real-time PCR using Peptidylprolyl Isomerase A gene (Ppia, Mm02342430-g1) as the housekeeping gene. PCRs were performed with the StepOnePlus™ Real-Time PCR System (Applied Biosystems, Courtaboeuf, France).

### 2.10. Statistical Analysis

Data were expressed as mean ± standard deviation of the mean (SD) and analyzed with the Prism 6.0 software (GraphPad, La Jolla, CA, USA). Comparisons between multiple groups were performed with the Kruskall-Wallis non-parametric ANOVA test followed by two-by-two comparisons with the Mann-Whitney U test, when a difference was detected. *p*-values ≤ 0.05 were considered significant.

## 3. Results

### 3.1. Physicochemical Characterization of Particles

**Figure 2:**
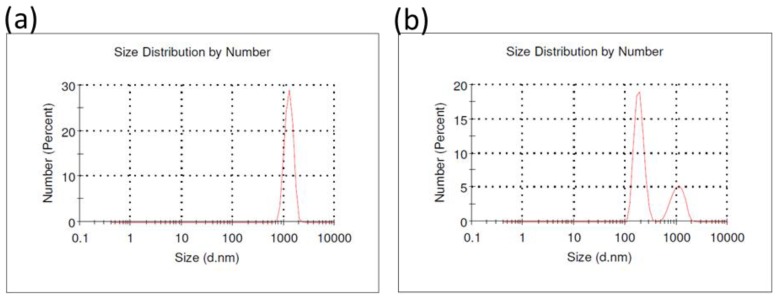
Size and number distribution of particle suspensions in HBSS used in the acute and subchronic exposure protocols. (**a**) FF solution (n = 3). (**b**) UFP solution (n = 3). FF solution revealed the presence of 1265 ± 239 nm particles. However, the UFP solution revealed the presence of two peaks. The first peak corresponded to ultrafine particles of 188.0 ± 39.9 nm. The second (1078 ± 305 nm) was correlated to particles aggregates formed in the samples.

The analysis of the particle size distribution by DLS revealed the presence of one peak at 1265 ± 239 nm in the FF solution. However, the analysis of the UFP solution indicated a bimodal distribution with two peaks at 188.0 ± 39.9 nm and 1078 ± 305 nm corresponding to the presence of ultrafine particles (71.9 %) and the formation of particle aggregates (28.1 %), respectively (Figure 2).

The analysis of the concentrations of trace elements showed that the FF and UFP samples had almost similar elemental compositions, with some variations in the degree of enrichment of certain elements. The FF contained high Mn and Zn levels characteristic of industrial influence, comparable to those already reported in the industrial region of Dunkirk [31,34,37,38]. The metal contents in UFP solution suggest the presence of large amounts of As, Ni, Pb and V, when compared to the concentrations found in the region, highlighting the industrial origin of these particles confirmed by the HCA. It must be noted that Na, Mg and K were not considered in the UFP solution analysis because these elements were initially present at high concentration in the HBSS buffer (Table 1). Interestingly, PAH concentrations appear to be quite similar in FF and UFP samples with a slight increase (10%) in their total amount in the FF solution (Table 2). In particular, BeP is greater in FF than in UFP. Similar observations have been reported by Saarnio et al. (2008) in six European urban cities (with 4 to 6 times higher values of BeP in PM_0.2–2.5_ than in PM_0.2_) [39]. This is probably due to a longer residence time in the atmosphere inducing an accumulation in the larger mode. Otherwise, there are many different combustion sources in the industrial and urban area of Dunkirk (North of France) that could preferably emit BeP in this fraction.

### 3.2. Internalization of Particles in Lungs

**Figure 3:**
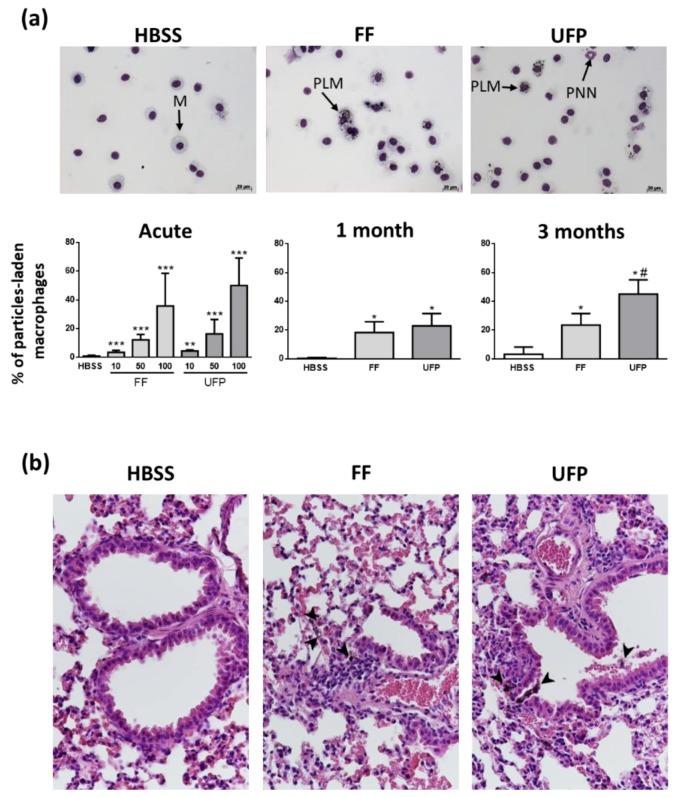
Internalization of particles in lungs. (**a**) Representative micrographs of BAL cells of mice exposed for three months and histogram showing BAL cell count of particle-laden macrophages; M = macrophage, PLM = particle-laden macrophage, PNN = Polynuclear Neutrophil. (**b**) Tissue micrographs of lung tissue (40×); Arrow heads point out particle-laden macrophages. * *p* ≤ 0.05 vs Control; ** *p* ≤ 0.01 vs Control; *** *p* ≤ 0.001 vs Control; # *p* ≤ 0.05 vs FF.

We observed aggregates of particles in the cytoplasm of macrophages present in the BAL from mice exposed to FF and UFP (Figure 3a). These data confirmed that the intranasally-administered particles were effectively delivered in mice lungs. The number of particle-laden macrophages increased in a dose-dependent manner in acutely-exposed mice (Figure 3a). After 1 and 3 months of exposure, the number of macrophages containing particles significantly increased with time in the exposed groups (Figure 3a). This increase was more visible in the UFP exposed animals. Similarly, particle-laden macrophages were also observed in lung tissue sections of the exposed mice, confirming the result obtained in the BAL samples (Figure 3b).

### 3.3. Inflammatory Cell Analysis in BAL

**Figure 4:**
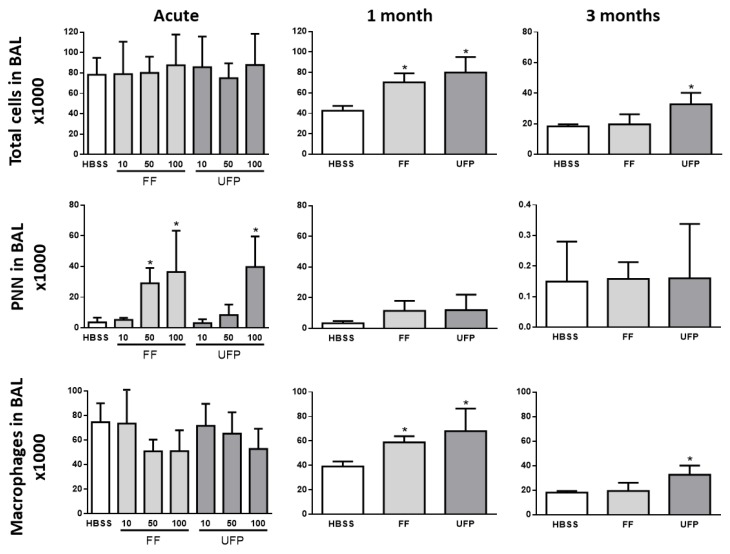
Inflammatory response analysis in BAL of mice exposed to FF and UFP. Histograms show BAL cell count in BAL of mice exposed for 24 h, 1 or 3 months: total cellularity, total number of PNN, total number of macrophages; n = 5 per condition; * *p* ≤ 0.05 vs Control; ** *p* ≤ 0.01 vs Control; *** *p* ≤ 0.001 vs Control; # *p* ≤ 0.05 vs FF.

BAL cell count is commonly used in diagnosis of lung inflammation and pulmonary diseases. The results of the cellular count in the BAL of mice exposed for 24 h neither show any effect of the particles on the total number of cells nor on the number of alveolar macrophages. On the other hand, a significant dose-dependent effect was observed on the number of neutrophils (Figure 4). With regard to the 1- and 3-month exposures, we observed an increase in the total number of cells correlated with an increase in the number of macrophages in the BAL of exposed mice. These cells were more increased in mice exposed to UFP compared to those exposed to FF (Figure 4). The number of PNN did not appear to be significantly affected by the 1- and 3-month exposures to particles.

### 3.4. Differential Expression of Inflammation-Related Genes in the Lung of Mice Exposed to FF or UFP

A set of genes related to inflammation (il-6, il-1b and il-10) and xenobiotic metabolism (Cyp1a1, Cyp1b1) was selected to confirm the interaction of the administered particles with pulmonary cells. The mRNA expression of the genes was measured by quantitative real time qPCR (Table 3). Only genes with a fold change above 1.5 were considered. After acute exposures, Cyp1a1 and Cyp1b1 increased significantly in mice exposed to 100 µg of FF or UFP. Cyp1a1 increased in both FF and UFP-exposed mice for 1 and 3 months, and Cyp1b1 expression was significantly induced only after 3-month exposures. Gene expression of the inflammatory cytokine il-6 increased significantly only in mice exposed to 100 µg UFP in the acute exposure. il-6 expression decreased in the FF and UFP-exposed groups in the 1-month exposure protocol; however, il-6 and il-10 expression increased after three months of exposure but only in mice exposed to UFP.

### 3.5. Lung Tissue Remodeling

**Figure 5:**
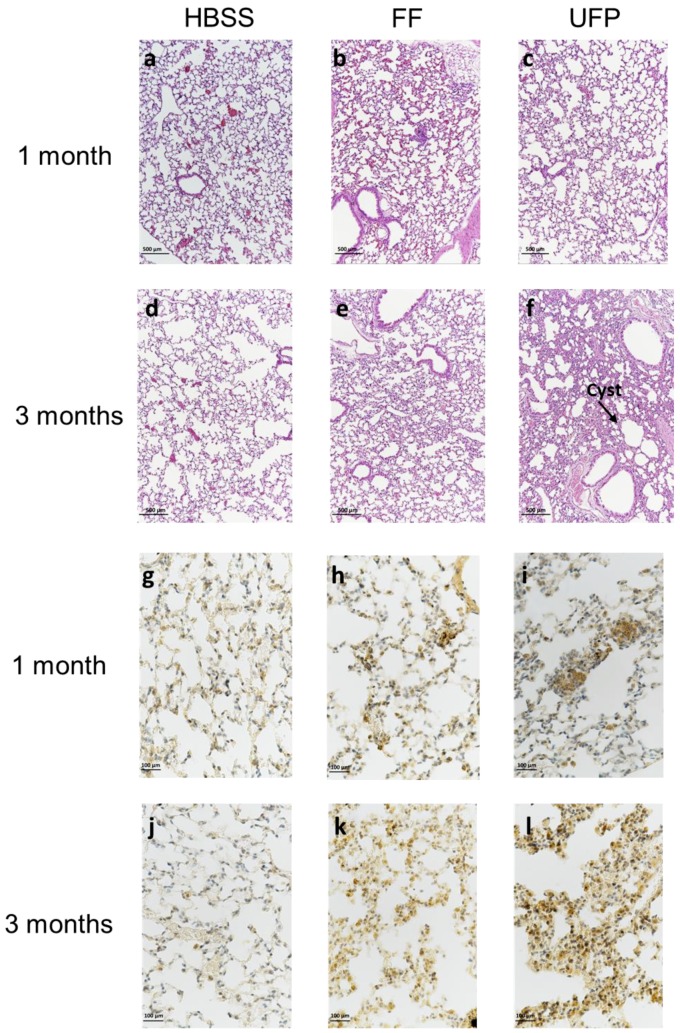
Lung tissue remodeling following exposure to FF or UFP after 1 or 3 months. (a–f) Lung histology (hematoxylin and eosin stain) showing alveolar wall thickening in mouse lungs; Cyst = cystic region; magnification 10×. (g–l) Immunohistochemical staining of mouse lungs; representative images of lung tissue sections stained for F4/80 (macrophages are stained brown); magnification 40×.

In order to assess the impact of UFP on lung inflammatory response, we performed a morphometric analysis of lung tissue sections in mice subchronically exposed to FF or UFP for 1 and 3 months. Inflammatory foci appeared in the peribronchiolar and alveolar areas, resulting in tissue damage (Figure 5a–f). The distribution of the lesions varied according to the size of the administered particles. They approximately spread over 8% and 18% of the overall lung surface in mice exposed to FF and UFP for 1 month, respectively (Table 4). For three-month exposures, they reached 35% and 47% of the total lung surface in mice exposed to FF and UFP, respectively. The injured areas consist of the presence of cystic regions characterized by slight enlargements of the airspaces in some loci (more marked in mice exposed to UFP for three months) and thickening of the alveolar walls of 7 µm to 14 µm in mice exposed for 1 month to particles, and 12 µm to 20 µm in mice exposed for three months. Sirius red staining of the lung histological sections failed to detect collagen fibers in the lesioned areas suggesting no evidence of fibrosis-like damages at these stages of exposure (data not shown). An anti-F4/80 staining of histological sections confirmed that the lung tissue remodeling consisted of alveolar macrophages infiltrations (Figure 5j–l). Interestingly, the number of macrophages in the injured areas was much higher in mice exposed for 1 month to UFP, compared to those exposed to FF. For the three-month exposures, the number of macrophages in the lesioned areas tended to decrease in mice exposed to FF, but it continued to dramatically increase in mice exposed to UFP (Table 4).

## 4. Discussion

Exposure to high levels of PM in the air leads to the exacerbation or development of different respiratory diseases [4,40]. PM toxicity is mainly linked to the development of inflammation in the respiratory system [41,42]. Compared to coarse and fine particles, little is known about the toxicity of UFP while a number of studies have suggested that they are more pathogenic than larger ones [19,43,44]. The current body of literature focuses on studying the toxicity of NM on health, while the long-term effect of exposure to UFP is not well assessed. In the present study, we assessed the effect of acute and repeated exposure to UFP on the respiratory system in a BALB/c mouse model. Another group of mice was exposed to the FF following the same exposure protocol in order to evaluate the outcomes resulting from exposure to the same dose in mass of this size fraction of particles. 

UFP and FF were collected from the same urban industrial site, near Dunkirk in Northern France. The physicochemical characterization of these particles was first performed in order to elucidate their origins and link their toxicity to their composition and size. The size of particles used in this study was verified by DLS, indicating the effectiveness of particles sampling. We then measured an important amount of specific metals in the collected particles, above the regional urban average, indicating that these particles were impacted by the local industries and the road traffic [31,34,45].

Several studies highlighted the deposition of intranasally administered particles in lungs of mouse models [46,47]. The presence of particle-laden macrophages both in lung tissues and BAL of exposed mice confirmed that FF and UFP administered intranasally could reach the deepest areas of lungs. This was also attested by the increase of gene expression of the P450 cytochrome Cyp1a1 in the lung of exposed mice. This gene is known to be induced by polycyclic aromatic hydrocarbons (PAH) such as those detected within the particles we collected [48,49]. Interestingly, we observed more particle-laden macrophages in the UFP-exposed animals. This might be due to the accumulation of a higher number of UFP in lungs. In addition to the number of particles, it has been also shown that particle size plays an important role in their immunotoxicity [50]. Indeed, smaller particles are eliminated less efficiently by macrophages and remain for an extended period of time in lungs, which may promote steady recruitment of more macrophages and induction of chronic inflammation, as shown in our experiments [50].

In the present study, we first investigated the inflammatory response in the BAL and lung tissue samples of exposed mice. PNN constitute the first line of defense against foreign invaders (hours to days). They induce an acute inflammatory response by accumulating at the damaged site, where they eliminate and neutralize the inducers of inflammation [51]. Interestingly, our BAL differential cell count showed an increase in PNN number after 24 hours of exposure with no effect on the number of macrophages. This result suggested that an acute inflammation developed immediately after the instillation of particles in lungs. According to the literature, a prominent culmination of PNN number has also been described after a single dose of PM_2.5_ [52]. When inflammatory lesions mature, neutrophils die via apoptosis and then the infiltration of a second cell type occurs. Thereafter, macrophages traffic to the tissue injury site and eliminate the apoptotic neutrophils, cell debris and the rest of the inducers [53]. In our experiment, a marked increase of the number of macrophages was observed in BAL after repeated exposures, while neutrophils were no longer present. This is consistent with the chronic inflammation mechanism above-described where macrophages are continuously activated and present in the inflamed tissue [53,54]. These macrophages are normally eliminated from the inflamed site by efferocytosis in order to end the inflammation and get back to homeostasis. If the inflammatory response is not resolved and its activation is not interrupted, the condition would evolve into chronic inflammation that may result in damages in the surrounding tissue and the development of respiratory chronic inflammatory diseases such as COPD [51,53]. In the present study, after three-month exposures, a significant increase in the number of macrophages in BAL persisted only in mice exposed to UFP. Consistently with the data obtained in BAL cell count, we observed higher macrophage infiltration in lung sections of UFP-exposed mice. Furthermore, after three-month exposures, we also noticed a greater peribronchiolar inflammation in UFP-exposed mice compared to those exposed to FF. Under normal conditions, inhaled particles are eliminated by the mucociliary escalator and macrophage clearance; however, when exposed chronically to particles, bronchiolar inflammatory response may occur [55]. The higher mRNA lung expression of il-6 in mice exposed to UFP for three months confirmed the existence of more severe inflammation. A significant increase in il-10 mRNA expression was also detected in this group of mice, which means that an anti-inflammatory response has occurred in order to resolve lung inflammation induced by UFP. il-10 is described to promote the clearance of recruited neutrophils in vivo, which is consistent with the decrease of PNN number in BAL with time [56]. We tried to quantify the protein expression of cytokines in the mouse BAL and lungs using different methods (ELISA, Luminex assays). However, we failed to obtain consistent results, especially in mice exposed to UFP. Interestingly, it has been previously described that interactions between UFP and some cytokines may create non-biological artifacts when measuring the concentrations of these cytokines in biological samples [57,58]. This would explain why we encountered difficulties while performing our immunoassays. Additional severe histological modifications were observed after particle exposure, especially in lungs of mice exposed to UFP for three months. It consisted of alveolar wall thickness due to macrophages infiltration in lungs. UFP are known to impair macrophage phagocytosis more than FF, therefore they are eliminated less efficiently allowing them to accumulate and spread in lungs [22]. The formation of thickened alveolar septa following exposure to PM has been described in previous studies. For example, Churg et al. (2003) reported marked airway wall thickening in lungs of women exposed to high levels of particulate pollution [41]. In the present paper, we demonstrated that the subchronic exposure to UFP is able to induce the formation of these lesions. These effects are also observed in lungs of patients with COPD [59]. Interestingly, we did not notice any difference between the effects of FF and UFP on the size of the observed alveolar wall thicknesses. However, the distribution of these lesions, reaching half of the lung surface, as well as macrophage infiltration were much more extensive in UFP-exposed mouse lungs. The formation of cysts characterized by slight enlargement of the airspaces was also observed in lungs of mice repeatedly exposed to particles. The long-term evolution of this type of damage could lead to emphysema-like lesions. The major risk factor for emphysema, is cigarette smoking [60]. However, recent epidemiological studies have demonstrated that long-term exposure to low concentrations of airborne particles could as well be associated to the development and exacerbation of emphysema [61,62]. In the present study, the cystic lesions were more marked in the mice repeatedly exposed to low doses of UFP for three months in comparison to those exposed to FFs in the same conditions. 

However, before concluding, some limitations about the dose we used should be considered. Considering a respiratory minute ventilation of about 50 mL/min for a mouse, an exposure to 10 µg of PM_2.5_ would mimic an ambient exposure to a particle concentration of about 139 µg/m^3^ of air for 24 h, which may approximately correspond to PM_2.5_ during a pollution peak in the north of France, according to the regional air quality monitoring network (no data are available for UFP concentrations). In addition, a similar level of PM_2.5_ (132 µg/m^3^) is reached in road tunnels during rush hours [63]. Under these unfavorable conditions of dispersion, UFP concentrations reach 46.5 µg/m^3^ which is only three times lower than those observed for PM_2.5_ in the same environment [63]. Therefore, the doses we tested are not so far from normal ambient exposure levels. In the present study, the FF and UFP were collected at the same urban-industrial site and displayed similar chemical characteristics. Therefore, since mice were exposed to the same mass concentration of FF and UFP, the greater effects of UFP observed could be attributed to their higher number and smaller size allowing them to reach more easily the deepest airways and accumulate in the alveolar spaces [64]. Indeed, compared to FF that dominates the PM_2.5_ mass, UFP correspond to a minor mass fraction while they represent in number 80% of the aerosol. Therefore, it might be more relevant to determine the doses according to the particles number, notably for particles with low mass. This concept is not usually used in the emerging toxicological studies [55,65,66,67]. Thus, a standardized method for calculating doses of UFP based on particle number concentration is needed and would be valuable in comparing these studies and building a consistent body of literature concerning the toxicity of UFP. 

## 5. Conclusions

Using an in vivo mouse model, we demonstrated that repeated exposure to UFP for three months leads to a severe chronic lung inflammation evidenced by alveolar wall thickening and massive macrophage infiltration. We report for the first time that the extent of pulmonary injuries is much more limited in mice exposed under the same conditions to fine particles collected on the same site. Better understanding of the toxicity and the long-term effects of UFP in lungs and other organs would encourage the concerned authorities to limit efficiently their emissions and reduce the health outcomes caused by high concentrations of these pollutants in the atmosphere. 

## Figures and Tables

**Table 1:** Elemental composition of FF and UFP quantified by ICP-MS. Values are the mean of measurements carried out on three sub-samples representative of the initial sample.

Element (µg/g)	FF	UFP
As	23.0	64.8
Ba	140.3	64.4
Be	0.4	0.3
Cd	16.9	19.3
Ce	8.5	6.3
Co	5.4	9.3
Cs	4.4	8.9
Cu	367.0	425.2
La	8.4	3.1
Mn	1350.4	582.2
Mo	25.6	36.7
Ni	98.7	199.7
Pb	399.4	541.9
Rb	26.3	44.1
Sb	47.1	49.3
Sn	49.6	111.1
Sr	91.8	49.3
Ti	3.0	5.7
Zn	4000.2	2460.7
Cr	97.3	120.7
V	75.0	196.3
Al	5252.2	3621.1
Ca	17,904.5	11,857.4
Fe	16,406.1	10,267.0
K	1240.3	30,368 *
Mg	6591.3	6344.8 *
Na	43,510.8	283,147 *
Si	9383.5	7568.1

* Values not considered in the analysis due to their high amount in HBSS.

**Table 2:** PAH composition of FF and UFP quantified by HPLC-Fluorimetry.

PAH (ng/mg)	FF	UFP
Fluoranthene (FLA)	5.1	4.3
Pyrene (PYR)	4.2	3.7
Benzo(c)phenanthrene (BcPHE)	0.4	0.2
Benzo(a)anthracene (BaA)	6.9	4.2
Chrysene (CHR)	6.8	2.8
5-Methylchrysene (5MCHR)	1.1	3.4
Benzo(e)pyrene (BeP)	9.9	0.8
Benzo(b)fluoranthene (BbF)	7.6	8.0
Benzo(j)fluoranthene (BjF)	4.7	9.9
Benzo(k)fluoranthene (BkF)	7.4	4.6
Benzo(a)pyrene (BaP)	3.8	5.3
Dibenzo(a,l)pyrene (DalP)	0.8	0.4
Dibenzo(a,h)anthracene (DahA)	2.6	1.5
Benzo(g,h,i)perylene (BghiP)	12.0	12.5
Indeno(1,2,3-c,d)pyrene (IP)	14.0	12.1
Dibenzo(a,e)pyrene (DaeP)	3.6	2.7
Anthanthrene (ANTH)	0.1	3.3
Coronene (COR)	2.9	4.5
**Total**	**94.0**	**84.0**

Values are the mean of measurements carried out on three sub-samples representative of the initial sample.

**Table 3:** Lung mRNA analysis of Cyp1a1, Cyp1b1, Il-6, Il-1b and il-10 in mice exposed to FF or UFP for 24 h, 1 month or 3 months.

	Acute Exposure					
	FF 10	FF 50	FF 100	UFP 10	UFP 50	UFP 100
***Cyp1a1***	0.8 ± 0.5	1.2 ± 0.6	1.7 ± 0.5 *	0.8 ± 0.5	1.4 ± 0.4	1.7 ±0.5 *
***Cyp1b1***	1.3 ± 0.3	1.6 ± 0.5	2.6 ± 1.6 **	0.9 ± 0.4	0.9 ± 0.2	2.2 ±1.0 *
***Il-6***	2.3 ± 2.5	1.3 ± 1.1	1.7 ± 1.9	1.8 ± 0.9	2.7 ±1.9	6.1 ±4.1 *
***Il-1b***	0.7 ± 0.3	1.0 ± 0.3	0.8 ± 0.1	1.3 ± 0.2	1.7 ± 0.8	2.7 ± 1.2
***Il-10***	1.1 ± 0.6	1.6 ± 0.5	1.0 ± 0.4	1.0 ± 0.7	1.6 ± 0.6	1.7 ± 0.8
	**1 Month**		**3 Months**			
	**FF 10**	**UFP 10**	**FF 10**	**UFP 10**		
***Cyp1a1***	7.8 ± 4.2 **	3.5 ± 2.6 **	5.6 ± 2.0 **	1.8 ± 0.6		
***Cyp1b1***	1..9 ± 0.2	1.6 ± 1.1	2.7 ± 0.9 *	3.9 ± 2.2 **		
***Il-6***	0.4 ± 0.2 **	0.4 ± 0.1 *	2.5 ± 1.6	7.8 ± 2.9 *		
***Il-1b***	1.1 ± 0.6	0.9 ± 0.2	0.7 ± 0.1	1.0 ± 0.3		
***Il-10***	0.4 ± 0.2	0.5 ± 0.1	2.2 ± 1.9	3.9 ± 1.0 *		

Data are presented as mean values and standard deviations. Gene expressions were calculated by the relative expression ratio of each gene to Ppia. n = 5; * *p* ≤ 0.05; ** *p* ≤ 0.01 vs control.

**Table 4:** Summary of histological lung changes following sub-chronic exposure to particles.

	1 Month			3 Months		
	HBSS	FF	UFP	HBSS	FF	UFP
**Peribronchiolar inflammation**	0 ± 0	0.7 ± 0.6	0.7 ± 0.6	0 ± 0	1 ± 0.0	1.7 ± 0.6
**Total macrophages/10 HPF**	45.3 ± 16.3	275 ± 81.8	342.7 ± 74.4	53 ± 12.0	211 ± 122.7	500 ± 213.7
**Cystic regions**	0 ± 0	1.3 ± 0.6	0.7 ± 0.6	0 ± 0	1.3 ± 0.6	1.7 ± 0.6
**Alveolar wall thickness**	0.7 ± 0.6	1.7 ± 0.6	1.7 ± 0.6	1.0 ± 0	1.7 ± 0.6	2.3 ± 0.6
**Lesioned parenchyma surface (mm^2^)**	0.6 ± 0.8	5.5 ± 1.0	9.8 ± 2.0	1.8 ± 0.8	21.9 ± 9.0	28.3 ± 3.8
**Overall lung surface (mm^2^)**	78.3 ± 4.3	66.3 ± 9.1	56.5 ± 8.4	64.8 ± 7.7	68.3 ± 14.0	61.7 ± 14.1
**Percentage of lesioned areas**	0.8 ± 0.9	8.3 ± 0.4	18 ± 6.6	2.7 ± 0.9	35 ± 21.2	47.3 ± 11.5

Peribronchiolar inflammation and cystic region scoring: None = 0, light = 1, moderate = 2, marked = 3, severe = 4. Total macrophages were counted in 10 high power fields (HPF) for each mouse. Alveolar wall thickness was determined by comparing the thickness of alveoli wall to the size of an erythrocyte (E): <1 E = 0; 1 to 2 E = 1; 3 to 5 E = 2; 6 to 10 E = 3; and >10 E = 4. The percentage of lesioned areas represents the distribution of thickened parenchyma surface to overall lung surface. HPF: high power field = 400.
